# Autism diagnosis as a social process

**DOI:** 10.1177/13623613211030392

**Published:** 2021-07-09

**Authors:** Jennie Hayes, Tamsin Ford, Rose McCabe, Ginny Russell

**Affiliations:** 1 University of Exeter, UK; 2 University of Cambridge, UK; 3 City, University of London, UK

**Keywords:** autism spectrum disorders, diagnosis, health services, policy, qualitative research

## Abstract

The diagnosis of autism can be challenging, particularly if an individual coming for assessment is considered to be near the diagnostic threshold. It is important to understand the experiences and challenges of diagnosis from the perspective of clinicians. In this study, 21 in-depth interviews were conducted with clinicians working in specialist autism assessment teams in adult and children’s services in England. Interviews were recorded and transcripts were analysed thematically. We identified four themes that represented how clinicians were frequently engaged with juggling their own professional understanding of what autism is with other factors such as the results of standardised tests and the views of patients and carers, in the context of limited resources: institutional pressure, making diagnosis make sense, seeing through an autism lens and just tools. The study illuminates the diagnostic process as a socially situated activity. We suggest that an examination of the benefits and drawbacks of assessment services specialising in autism only, the resources they require to operate effectively, and how they operate in the context of wider health services would be appropriate and timely.

**Lay abstract:**

When a child or adult is referred for an autism diagnosis, clinicians from different backgrounds work together to make a diagnostic decision. A few studies have asked clinicians in interview how they feel about diagnosis and what the challenges are. We interviewed clinicians in child and adult assessment services in England, and from different professional backgrounds, about the challenges of autism diagnosis and the factors that might influence the assessment process. We found that there were a number of challenges in autism diagnosis, especially when someone coming for diagnosis was considered to be near the diagnostic threshold. Clinicians told us that making a diagnosis was like creating a ‘narrative’: looking at many different factors that told a story about a person, rather than just looking at the results of diagnostic tests. Clinicians do not always agree with the results of those tests and have to use their specialist clinical judgement to make decisions. Clinicians were concerned about the amount of time people have to wait for an autism assessment, and the resulting pressure on the assessment process. The findings of this work can help us to understand how diagnosis happens and consider ways in which it can be improved for adults, children and families coming for assessment, as well as clinicians.

## Background

There is a growing body of work examining how parents and adults coming for an autism diagnosis experience the diagnostic process (e.g. Crane et al., 2016; Divan et al., 2012; Jacobs, Hens, et al., 2019; Jones et al., 2014; Osborne & Reed, 2008; Russell & Norwich, 2012). However, less is known about clinicians’ experience of the diagnostic process and the challenges they might face in their work.

Autism assessment is based on diagnostic criteria outlined in the *Diagnostic and Statistical Manual of Mental Disorders* (DSM) and the *International Classification of Diseases* (ICD) (American Psychiatric Association [APA], 2013; World Health Organization [WHO], 1993). Autism is classified as a neurodevelopmental disorder and is diagnosed when there are persistent patterns of difficulty in social communication and social interaction across multiple contexts, combined with restricted and repetitive patterns of behaviour, interests or activities (APA, 2013). Symptoms must be present in the early developmental period and must cause significant impairment in social, occupational or other areas of functioning (APA, 2013).

A comprehensive assessment involves considering core autism signs and symptoms as well as developmental history, behavioural difficulties, functioning at home, education or employment, consideration of differential diagnoses or coexisting conditions and hyper- or hypo-sensory sensitivities and attention to detail (National Institute for Health and Care Excellence [NICE], 2012). This is achieved through direct observation, interview, consideration of documentary evidence and, where appropriate, utilising a formal assessment tool. A range of diagnostic tools are utilised in practice to enable observation of behaviours, as well as gain an understanding of the patient/family concerns, experiences and history, focussing on DSM/ICD criteria (NICE, 2011, 2012). Some of the most commonly used tools include the Autism Diagnostic Observation Schedule (ADOS) (Lord et al., 2000) and a clinical interview tool such as the Developmental, Dimensional and Diagnostic Interview (3Di) or The Autism Diagnostic Interview–Revised (ADI-R) (Lord et al., 1994; Skuse et al., 2004).

The diagnosis of autism poses particular challenges for healthcare practitioners: there are no biomarkers regularly utilised in diagnostic tests (Vllasaliu et al., 2016) and the condition represents a heterogeneous group of disorders, with wide ranging levels of severity and symptom expression. Symptoms that are common to autism may occur with other conditions (Huerta & Lord, 2012), leading to ambiguity when observing behaviours, and research also suggests that diagnostic procedures are not consistent across practice (NICE, 2012). The process of diagnosis, therefore, is complex and multi-faceted, and can be particularly challenging when cases are considered ‘borderline’ or where there are coexisting conditions.

Despite the introduction of national autism strategies throughout the United Kingdom since 2008, the mechanisms for meeting diagnostic needs have failed to meet the level of demand, with targets for assessment timescales in children’s services routinely being missed (British Medical Association, 2019). With an increased number of people coming for assessment, therefore, UK assessment services are under significant pressure.

Several studies exploring clinicians’ views of autism assessment report that clinicians express concern about stretched resources and increasing caseloads (Crane et al., 2018; Rogers et al., 2016; Rutherford et al., 2016; Skellern et al., 2005; Ward et al., 2016). Overall, studies suggest a number of tensions in the diagnosis of autism.

Some studies identify a pressure to diagnose to enable access to services (e.g. see Jacobs, Steyaert, et al., 2019; Rogers et al., 2016; Skellern et al., 2005; Taylor et al., 2016). Two Australian studies found that clinicians had diagnosed autism, even when uncertain, to enable access to services or financial support (Skellern et al., 2005; Taylor et al., 2016). In the United Kingdom, a survey of clinicians involved in autism assessment of both children and adults found that 76% of respondents had ‘upgraded’ a diagnosis to autism when faced with ‘unclear presentation or patients failing to meet criteria on diagnostic tools’ (Rogers et al., 2016, p. 829). A Belgian interview study found that clinicians can feel ‘coerced’ to make a diagnosis to trigger appropriate services, thereby being constrained by the institutional necessity to diagnose, rather than reflect ‘generic everyday difficulties’ (Jacobs et al., 2018, p. 7).

These studies suggest that, in the context of concerns about limited resources and increasing caseloads, clinicians find pragmatic ways to deviate from standardisation when there is uncertainty and when the system is perceived not to meet patient needs.

In psychiatric diagnosis, clinicians adopt a holistic ‘bio-psycho-social’ model (Engel, 1977), meaning that a broad range of environmental, psychological, biological and social risk factors are considered. A thematic review of qualitative studies examining the conceptualisation of autism by parents and clinicians found that despite this bio-psycho-social approach in clinical practice, clinicians may tend to take a more bio-medical approach when communicating with patients (Jacobs, Hens, et al., 2019). The conceptualisation of autism as primarily a medical disease to be prevented, cured or treated (Russell & Norwich, 2012) or as a psycho-socially influenced condition impacts on the relationship between clinician and family, the perceived optimum outcome, as well as informing perceptions of ‘normal’ behaviours and development, including the identity of parents and child (Jacobs, Hens, et al., 2019). It is suggested that considering the different understandings of autism that different stakeholders may have can enhance the clinician–patient–family ‘alliance’ (Jacobs, Hens, et al., 2019). One study exploring professionals’ views in Singapore found that the quality of the relationship between parents and clinicians during assessment was important in reducing parental stress (Moh & Magiati, 2012).

Another challenge in diagnostics is the difference in the way patients and clinicians interpret and understand symptoms as ‘autistic’ or not, leading to patient–professional tension. An interview study with parents, autistic adults and clinicians in the United Kingdom found that there is a tension between patient/family and professional expertise, with parents/adults coming for diagnosis contesting the expertise of clinicians if they believed they were autistic (Crane et al., 2018). This can contribute to ‘barriers to satisfaction with the diagnostic process’ and concerns from clinicians that patient needs would not be met appropriately (Crane et al., 2018, p. 3766). This can also include inter-professional tension where professional knowledge of autism is contested between, for example, clinicians and teachers, as well as parents (Crane et al., 2018). Different understandings of autism, therefore, can contribute to the challenges of assessment for the clinician.

This study draws on the sociology of diagnosis, which argues that diagnosis cannot be separated from wider influences of human agency and deliberation (Jutel, 2011). A sociology of diagnosis approach challenges the taken-for-granted fit of diagnostic categories to their conditions and instead considers them as socially framed and shaped by wider social forces and interaction (Brown, 1995). We were, therefore, interested in how the process of autism assessment itself may impact on decision-making. The studies we have reviewed here demonstrate how diagnosis is, at times, shaped by a tension between meeting criteria and/or meeting patient needs; by the complexity of biological, psychological and social factors in the assessment process and through the need to resolve different understandings of the condition. Clinicians have to ‘find’ autism in an individual, and yet, this individual’s behaviour, and the assessment of it, is in itself a social process, shaped by institutional forces, locally available resources and expertise, the interaction between families and clinicians, and between clinicians themselves. This study considers the tensions in the autism assessment process that are faced by clinicians through 21 in-depth interviews with clinicians working in specialist autism assessment teams in adult and children’s services in England.

## Method

### Participants

Interview participants were drawn from four autism assessment teams involved in a wider observation study examining clinician interaction during assessment meetings (Hayes et al., 2020, 2021). Recruitment of teams was undertaken from an open call to a list of clinician contacts drawn from the Internet and via the National Institute for Health Research (NIHR) Clinical Research Network. All teams were located in England and were National Health Service (NHS) providers. Two teams specialised in adult assessment and two in the assessment of children and young people (C&YP). One team also specialised in diagnosing attention deficit hyperactivity disorder (ADHD). Sample size and participant selection for interview were determined by the number of clinicians involved in these teams, as only clinicians involved in the observation study were included. All regular attenders at the observed assessment team meetings were invited to participate in the interview study. A list of clinicians, their role, gender and age range is presented in Table 1. Specific data on race/ethnicity and socioeconomic status were not recorded.

**Table 1.** Characteristics of interview participants.

	Participant characteristics	
Role type	Consultant psychiatrists	4
	Clinical psychologists	6
	Educational psychologists	1
	Speech and language therapists	1
	Occupational therapists	1
	Senior/team/operational managers *(with specialist autism or social work clinical background)*	3
Team	Adult assessment	7
	Child and adolescent assessment	6
	Adolescent assessment (14+)	3
Demographics	Age range	30–60
	Years practised as a clinician	2–30
	Years practised autism diagnosis	1–12
	Female	13
	Male	3

### Data collection

A total of 21 interviews were conducted: 16 were individual semi-structured interviews as described below and five were structured interviews with key personnel, asking specific questions about local organisational structure, team make-up, referral and assessment processes. Interviews took place over a period of 11 months during 2017–2018 (see Supplemental Appendix 1 for interview topic guide).

Interviews took place after observation and audio-recording of the assessment meetings. A semi-structured interview framework was used for the first set of interviews which allowed for variation in interview depending on the interviewee’s experiences. The topic guide had three parts: questions around the diagnostic process, questions about diagnostic tools, and a section where specific cases were discussed and, where possible, audio extracts from meetings were played for clinician reflection using a method called Tape-Assisted Recall (TAR). In TAR, recordings are used to prompt relevant discussion about the topic in question (Elliott, 1986). Although commonly used in studying therapeutic relationships and patient/clinician consultations (Baker et al., 2019; Cape et al., 2010; Elliott, 1986; Elliott & Shapiro, 1988), it was anticipated that this method, utilising audio recordings from assessment meetings at which the clinician had been present, would provide a useful prompt to discuss issues arising from specific cases. Selected audio extracts from meetings were played during the interview, and clinicians were asked for their reflections. Transcripts were also available. In a small number of cases, it was not possible to play sound files for technical or practical reasons. In these cases, detailed notes or a transcript were used as the basis for discussion. Criteria for selecting audio extracts were as follows: (1) the interview participant had been present at the assessment discussion and (2) there had been an expression of uncertainty in the assessment discussion about whether the patient met diagnostic criteria. This was intended to generate reflection on some challenges of autism diagnosis.

### Data analysis

Interviews were audio-recorded and transcribed verbatim. A thematic analysis approach was used to identify patterns and themes within the data (Braun & Clarke, 2006). Inductive analysis followed Braun and Clarke’s (2006) framework for thematic analysis: transcription and familiarisation, generating initial codes, collating codes into potential themes, reviewing themes, defining and naming themes, and refining analysis through writing up. Codes and themes were identified on paper using colour-coded highlighters; exploration of groupings of codes was undertaken with mapping and index cards. Ongoing analysis was informed by sharing data at data analysis sessions and with co-authors.

### Note on terminology and anonymity

We acknowledge that there are different preferences for naming the condition; however, we use the term ‘autism’ throughout to embrace the spectrum of conditions as currently defined in the *Diagnostic and Statistical Manual of Mental Disorders* (5th ed.; DSM-5; APA, 2013) and the *International Statistical Classification of Diseases and Related Health Problems–Tenth Edition and Eleventh Edition* (ICD-10 and ICD-11; WHO, 1993, 2018). We use the term ‘patient’ for all children and adults coming for diagnosis, acknowledging the ‘medical’ nature of the setting in which people are discussed and ‘clinician’ to encompass all healthcare professionals included in the study. To preserve anonymity, we have not attributed quotes to individual clinicians as they could be identified by role.

### Community involvement

The study was part of a wider research project with an advisory board comprising autistic people, parents of autistic children, specialist clinicians and academics. The study was devised and revised after direct consultation with clinicians.

## Results

**Figure 1.**
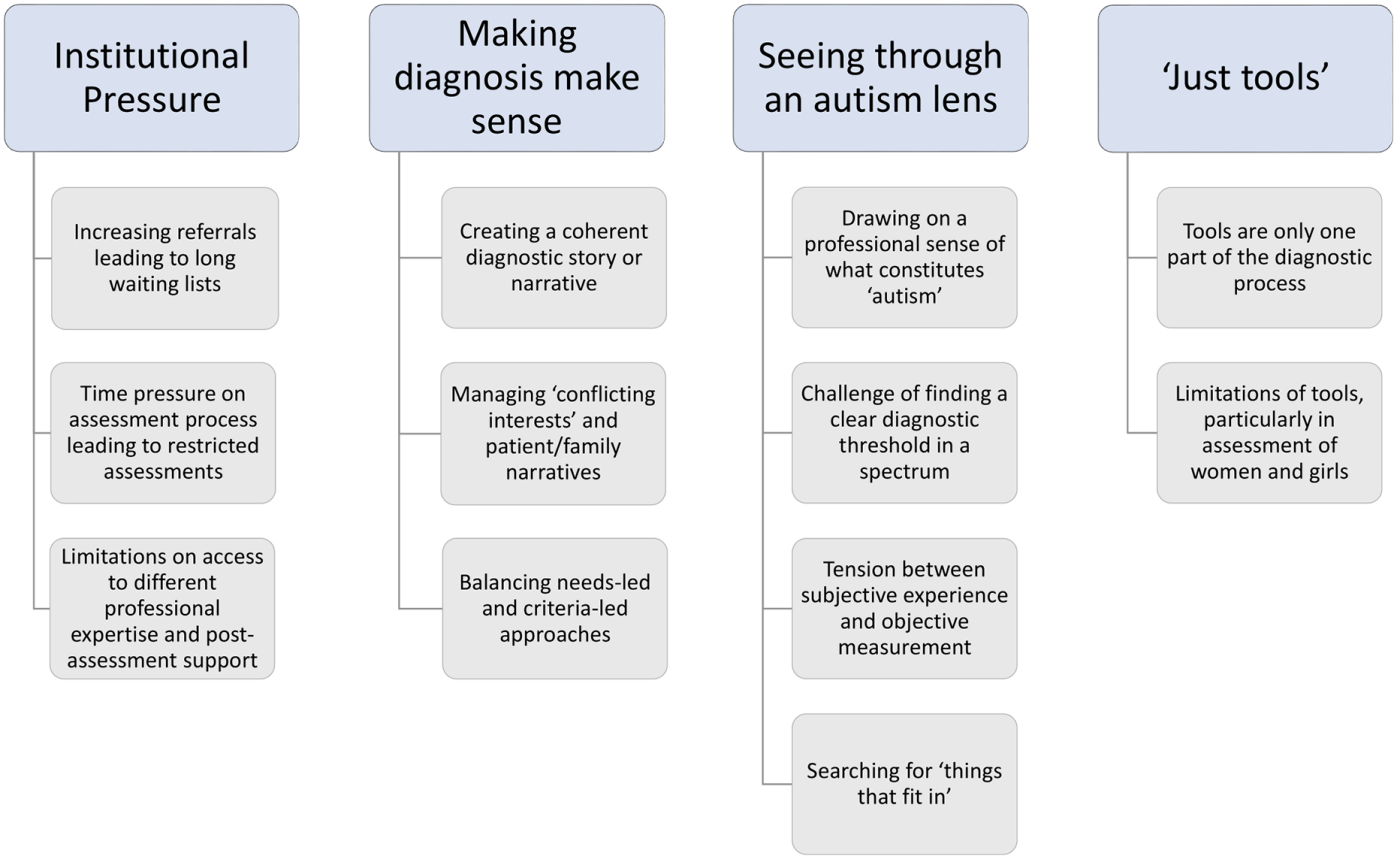
Themes and subthemes discussed by clinicians involved in autism assessment.

Four main inter-related themes were identified from the interview data: institutional pressure, making diagnosis make sense, seeing through an autism lens and ‘just tools’ (see Figure 1).

### Theme 1: institutional pressure

Across adult and children’s teams, clinicians were concerned about how the institutional limitations impacted on their ability to diagnose in a way they considered to be ideal. This concern was primarily related to increasing referrals leading to long waiting lists, contributing to pressure on the amount of time allowed for assessment. However, clinicians also discussed related concerns about the breadth of assessment possible within the current system.

The pressure of waiting lists on the assessment teams begins at referral:

No matter how hard we’ve worked over the last 7 or 8 years, and resources have been put at it and we’ve looked at how we can work more efficiently, we just can’t keep up with the volume of referrals.

This clinician expressed a view that some referrals were just not appropriate for the autism pathway and that this contributed to ‘too many’ children coming for assessment:

Far too many children are coming in and the reason for that is mixed, but . . . you can have challenging families trying to work out what the difficulties are for their child and they’re pushing, pushing, pushing, and some of the paediatricians have actually said ‘well we’ve put them into the pathway because it gets the parents off our back and we know they’re going to get a good thorough assessment’ . . .

Some considered that the pressure of long waiting lists compromised what they considered to be best practice, with time pressure on the assessment process leading to restricted assessments, either through lack of time for deep discussion, or a reduction in the number of observations in different settings that were possible:

I mean there’s still a waiting list . . . a minimum of a year . . . that pressure to get people through the system, which is obviously important, I think sometimes has watered down the time for good team discussion.

One team member expressed a frustration about being unable to undertake additional assessments (e.g. cognitive assessment) that would be helpful for the patient but not essential for a diagnosis, thereby challenging the ‘narrow focus’ of autism assessment in the context of long waiting lists:

We’re commissioned to look at the assessment of autism and that’s it. Every young person we do an additional assessment for is another one waiting a bit longer.

This pressure on services has also impacted, in children’s services, on the number of assessments which can include a full school observation:

I guess pressures of time, the school observation is not done as much now, . . . that might help partly explain why the sheer number getting the diagnosis has gone up, because we don’t have that observational context . . . where the child will be behaving in as much of a normal way.

Here, the clinician considers that, because of increased numbers coming for assessment, there is less time to do full school observation, the lack of which they suggest is likely to contribute to increasing diagnostic rates. This clinician went on to say:

In the last probably 18 months/two years, because of the pressures on the number of children in the system and trying to get the waiting period down, that robustness (of assessment) has been compromised.

While other clinicians did not go as far as suggesting the process itself is compromised, there was a strong sense throughout that the background of limited resources, a backlog of people waiting for assessment (‘our waiting list at the moment is 18 months’) and the resulting pressures on patients and families, in both adult and children’s services, were a factors that put a strain on their diagnostic processes and sometimes limited the length of time for assessment.

Finally, the breadth of assessment was further compromised by limitations on access to different professional expertise for specialist assessment (‘Unfortunately, there isn’t a speech and language therapist within our directorate, or somebody that we could access’) and difficulties with providing post-assessment support (‘because we are an assessment only service . . . we can only do so much support after the diagnosis, and there is a gap there for sure’). Overall, therefore, long waiting lists, time and resource restrictions and the limited role of the assessment team were considered to be problematic.

### Theme 2: making diagnosis make sense

Clinicians understood diagnosis in terms of creating coherent diagnostic story or narrative, weaving together the results of a number of different facets of the assessment process. They generally took a nuanced approach to assessment and diagnosis, with an appreciation of the meaning of the diagnostic label for their patients and their families. However, they were also strongly aware of the need to manage ‘conflicting interests’ and patient/family narratives: the knowledge that patients and families might not share their understanding of the difficulties or agree with the outcome of the assessment.

Clinicians generally understood assessment as a process that ‘makes sense’ of a person’s lived experience:

Diagnosis is about narrative actually, creating a narrative that makes sense of people’s experiences.

One clinician considered that both clinicians and patients construct narratives about behaviours that are selective, in service of a sense-making narrative:

We all look to make a coherent narrative of how we see things and how we see the world, so it’s the same if a parent was actively not wanting a diagnosis, they’d discount things that didn’t fit with the idea of . . . you’ve got a view of how things are so you discount the things that go against that . . .

The ‘understanding’ that comes via diagnosis was seen to be important in relation to the help that is then given (‘the whole point of diagnosis is . . . understanding how to support someone’) and this was linked to making a decision that was ‘accurate’ or ‘doing the right thing’. There were different positions on this, balancing needs-led and criteria-led approaches. One clinician felt strongly that it was important to consider the desires and identity of the patient, particularly as they were entering adulthood (‘if people don’t wish to identify with a diagnosis . . . we shouldn’t force them to’). Others felt there could be a case for diagnosis if it would help the patient/family – taking a needs-led approach – especially in threshold cases (‘especially with people who are borderline . . . we think is it going to help them’). Some considered that a stricter recourse to diagnostic criteria, a ‘criteria-led approach’, can help in tricky cases or when there is perceived pressure from families to diagnose autism (‘it really does come down to the diagnostic criteria’).

Clinicians were highly aware of how a diagnosis may impact on the patient/family, and assessed the potential consequences of diagnosis, both in terms of access to support and, as above, to wider issues of identity, for example. In some cases clinicians acknowledged the way in which the desire of the patient/family could, on occasion, influence the assessment (‘we gave mum too much weight’) but were also clear that it was possible to resist the patient/family view when necessary (‘I think people are identifying with it . . . it doesn’t really overly impact my decision’). Clinicians discussed the difficulties of managing the expectations of patients and families who are ‘invested’ in an autism diagnosis (‘it’s the only thing that makes sense of me’) and those who appeared unaware that autism might be a possibility (‘they were just like ‘yeah, yeah’, and they say everything’s fine . . . no he doesn’t have concern in these areas . . .’).

Overall, there was a strong sense that patients and families are considered to be active agents in the diagnostic process. For example, clinicians were aware that adults or families coming for assessment could research autism symptoms to prepare for assessment or even rehearse (‘they could go on the Internet and read the sorts of criteria . . . so they will say the right things’) which could ‘skew’ the assessment, or disrupt the clinical narrative. This could extend to individuals repeatedly seeking assessment despite a negative diagnosis:

It was said ‘no they’re not autistic’, and the parents didn’t agree . . . so they took them to (another diagnostic service) . . . and she said ‘yes I think there’s something there’ . . . and they got the diagnosis . . . two professionals say no and then two professionals say yes.

To conclude, clinicians considered autism diagnosis as a way to understand and explain particular behaviours. However, they acknowledged that patients and families do not always share that understanding or explanation and that this can sometimes impact on assessment. They expressed a need to manage the extensive expertise and specialist knowledge of patients and families as well as their expectations. Overall, this reflected a tension familiar in the autism world of ‘who is the expert?’ (Crane et al., 2018): the clinical team with the power to assign a diagnosis, the family or carer of the patient, or the person with lived experience of autism?

In certain cases people will leave here . . . perhaps more confused than when they arrived because they felt they had an understanding of themselves, and we have a different perspective to them.

### Theme 3: seeing through an autism lens

When different elements of the assessment do not concur, clinicians resolve this, at least in part, by drawing on a professional sense of what constitutes autism. The ‘autism lens’ used by the team enables the clinician to recognise the ambiguity of symptoms while at the same time, utilise their individual clinical experience and informed judgement to guide decision-making, particularly at times of uncertainty. This professional ‘lens’ is akin to Foucault’s (1973) ‘medical gaze’: a professional authority afforded through the clinic. For example, one clinician said,

I do feel now when I see people that I usually know within five minutes, most people, with autism, like I can kind of tell already.

This sense of ‘feel’, as described here, can help recognition (‘for us, they (people with autism) feel very different’) and can guide what they, as experienced clinicians, might generally expect in assessment, their ‘normative experience’:

I wouldn’t say it’s like a psychoanalytic kind of how they make me feel . . . I think it’s more about . . . a social understanding of what you’d expect the person to manage.

While one clinician considered that diagnosis can be straightforward (‘it’s very easy to make a diagnosis if someone is very clearly not autistic or very clearly autistic’), there was a general sense that diagnosis can be ‘tricky’ or uncertain when there are potential co-occurring conditions, when tests do not agree or in what are considered borderline or threshold cases. Clinicians voiced the challenge of finding a clear diagnostic threshold in a spectrum:

We have to give a binary outcome in this clinic, but in reality, as we know . . . autism can be viewed as more of a spectrum.

There were cases where people ‘don’t quite meet criteria’, where behaviours may be ambiguous, or individuals may appear to have few difficulties (‘superficially, if you just met her casually, maybe it wouldn’t be an issue . . . she wouldn’t necessarily score’ (on the standardised diagnostic test)). One clinician noted that ‘people can show traits of autism without necessarily meeting the full diagnosis’ and another that ‘I don’t think we can be that black and white about everybody’.

Clinicians expressed tension between subjective experience and objective measurement: reference to standardised diagnostic criteria is considered to be important, but subjective experience should not be overlooked:

I don’t want to prioritise my subjective experience too highly . . . but I also don’t want to discount the fact, how it felt, because that’s a crucial part . . .

However, even diagnostic criteria themselves require interpretation. One clinician said that ‘someone could meet that (ICD) criteria without necessarily being obviously autistic’: the specialist lens of the clinician is necessary to determine the presence of autism or not. The lens – a particular and specialist way of seeing and understanding – is shaped by clinical training, experience and background (Goodwin, 1994): one clinician considered that a lack of professional experience in one area could mean that one perspective ‘wouldn’t necessarily be given the same kind of weight . . . this very similar set of symptoms could be understood in a slightly different way’.

The ‘lens’ is thrown into sharper focus when the team’s role is narrow. The purpose of the specialist autism assessment team is to provide a clear route for autism diagnosis, with other health difficulties typically having different pathways. One clinician was troubled with how the institutional framework of autism diagnosis encouraged all parties to see behaviours ‘through an autism lens’ and ‘search for things that fit in’:

You come along with this concept of what autism is and everything that you look at becomes filtered through that lens . . . a search for things that fit in . . . that does sometimes close down thinking.

This lens or ‘professional vision’ (Goodwin, 1994) enables a particular kind of gaze which authorises the clinician, when uncertain, to make a judgement about behaviours in an assessment context. Clinicians acknowledge this as subjective (‘(diagnosis) is not a precise art, it’s a difficult thing; it can be quite subjective at times’). Clinicians, therefore, recognised the balance of subjectivity and objectivity inherent in the assessment process but had different ways of balancing the significance of standardised diagnostic criteria, their individual intuitive responses to their patient and the dilemmas inherent in interpreting behaviours one way or another.

### Theme 4: ‘just tools’

Clinicians frequently referred to the limitations of standardised diagnostic tools, particularly the ADOS. In line with diagnostic guidelines (NICE, 2011, 2012), this included an understanding that tools are only one part of the diagnostic process, and should not be relied upon alone; as well as acknowledging the limitations of tools, particularly in the assessment of women and girls. However, the ADOS was seen as a useful tool to provide a qualitative focus on the interaction as well as a numerical score: ‘the ADOS is the biggest tool that I use, and it is really helpful . . . it is subjective but it’s objective as well in a lot of ways’.

Generally, diagnostic criteria, and the tools that interpret them, were thought to be helpful but with constraints:

Sometimes, they can be a bit of a straight-jacket that we’re . . . Yes, tick, tick, tick, tick, and you’ve got this tick box exercise rather than this ‘what’s the whole picture?’ and so I guess it’s a judgement around whether it’s going to support that discussion or whether actually it might hamper it a bit.

Diagnostic tools were considered to be only one part of the diagnostic process. For example, one clinician said, ‘the ADOS and the 3Di are just tools to lead us somewhere, and I don’t think it would be ethical if you put everything on a number’.

This means that a diagnosis has to take into account a range of elements, including the patient history and observations, so that ‘we’re all agreed that we’re happy to make a diagnosis in adults, even if they score below the threshold on the ADOS’. Overall, the ADOS was considered to be ‘partial and . . . a snapshot and not definitive’.

Clinicians expressed difficulties in using the ADOS when assessing particular groups due to limitations in its design:

Cut-offs are helpful but not an absolute, concrete cut-off, if you like, because actually for a start the ADOS was normed predominantly on American men, so there are some cultural differences.

Diagnosing women and girls was considered to be particularly problematic, with a common view that they have learned to manage their difficulties and therefore developed effective social skills:

. . . the problem with using it in adults, and particularly in women . . . when you do the ADOS, they don’t score, because they mask their difficulties and they’ve learnt how to behave and how to interact and what you should do.

In addition, diagnostic tools were considered to have limitations if there were likely co-conditions:

. . . if somebody has a learning disability sometimes they score higher on certain areas just because of the nature of some of the questions in the ADI . . . when there’s maybe a co-occurring mental health difficulty or there’s a learning disability, or a brain injury, and actually it’s quite difficult sometimes to unpick some of those from the developmental history . . .

Clinicians considered, therefore, that going against the ‘score’ was either related to the inadequacy of the tool to pick up difficulties, for example, ‘female presentation’, due to skilled coping mechanisms on the part of the patient; a failure of the tool to differentiate between autism and learning disability or anxiety, for example; or simply one mechanism for assessment in the context of a wider view of assessment processes. Therefore, rather than the diagnosis of autism being difficult to attach to the criteria, the tools themselves failed (at times) in their ability to ‘find’ autism and therefore had to be over-ruled by the clinicians’ ability to interpret behaviours in a different way, that is to ‘see it differently’. This position assumes that autism can be ‘found’ independently of the tests that measure it.

## Discussion

This study concurred with others that show the challenges of adequately meeting patient needs within the diagnostic process due to increasing caseloads, the drive for diagnosis to act as an explanatory framework and the need to manage uncertainty. While it was evident that clinicians take into account a range of environmental, psychological, biological and social risk factors, we were unable to compare different conceptualisations of autism as we did not interview parents or patients. However, it was clear that clinicians were aware of the potential for misalignment of views between patients, families and clinicians (Crane et al., 2018; Jacobs et al., 2018; Skellern et al., 2005). This, in itself, contributes to the tensions in assessment.

Our themes demonstrate the way in which pressure on services ‘trouble’ clinicians engaged in life-changing decisions, and offer some insight into how they carve diagnostic decisions out of complex, social, human situations, balancing the needs of the patient to receive appropriate support, with meeting diagnostic criteria to create an appropriate and meaningful diagnostic narrative. Clinicians’ use of diagnostic tools is both pragmatic and cautious, reflecting diagnostic guidelines which advise utilising diagnostic tools in the context of other assessments. They also adopt current thinking about the ADOS tool lacking sensitivity (the ability of a test to correctly identify those with the condition - a true-positive rate), particularly when diagnosing women and girls (see Lai et al., 2015; Loomes et al., 2017). Despite clinical guidelines, standardised diagnostic criteria and a range of autism-specific tools, diagnosing autism is not always an easy task. This is where professional judgement comes to the fore, which asserts the special knowledge of the clinician to assign meaning to certain behaviours, even if those behaviours are ambiguous.

Clinicians’ ability to pull apart a complex tangle of social behaviours and contradictions that make up autism relates to what Goodwin (1994) terms ‘professional vision’. Clinicians, as a community, have the ‘power to legitimately see, constitute, and articulate’ (Goodwin, 1994, p. 626) what autism is, in a way that patients do not. The perspectives and interests of patients and families differ from, and may align or conflict with, those of clinicians. There may be disagreement between clinicians, patients and families about the meaning of a person’s behaviours, the diagnostic outcome, as well as the perceived potential impact of a diagnosis in relation to access to support and resources, for example. However, the ability to ‘see’ autism, remains a socially situated activity, confined to the context of the professional assessment process. As explained by Pilnick and James (2013, p. 90), ‘being able to see a meaningful event is not a transparent psychological process, but is instead a socially situated activity’. The practical application of professional vision is not straightforward or singular. Clinicians too disagree, between themselves, and with other professionals, and individual clinicians can encounter challenges in integrating conflicting views from their training and subsequent clinical experience (Jacobs, Steyaert, et al., 2019).

**Figure 2.**
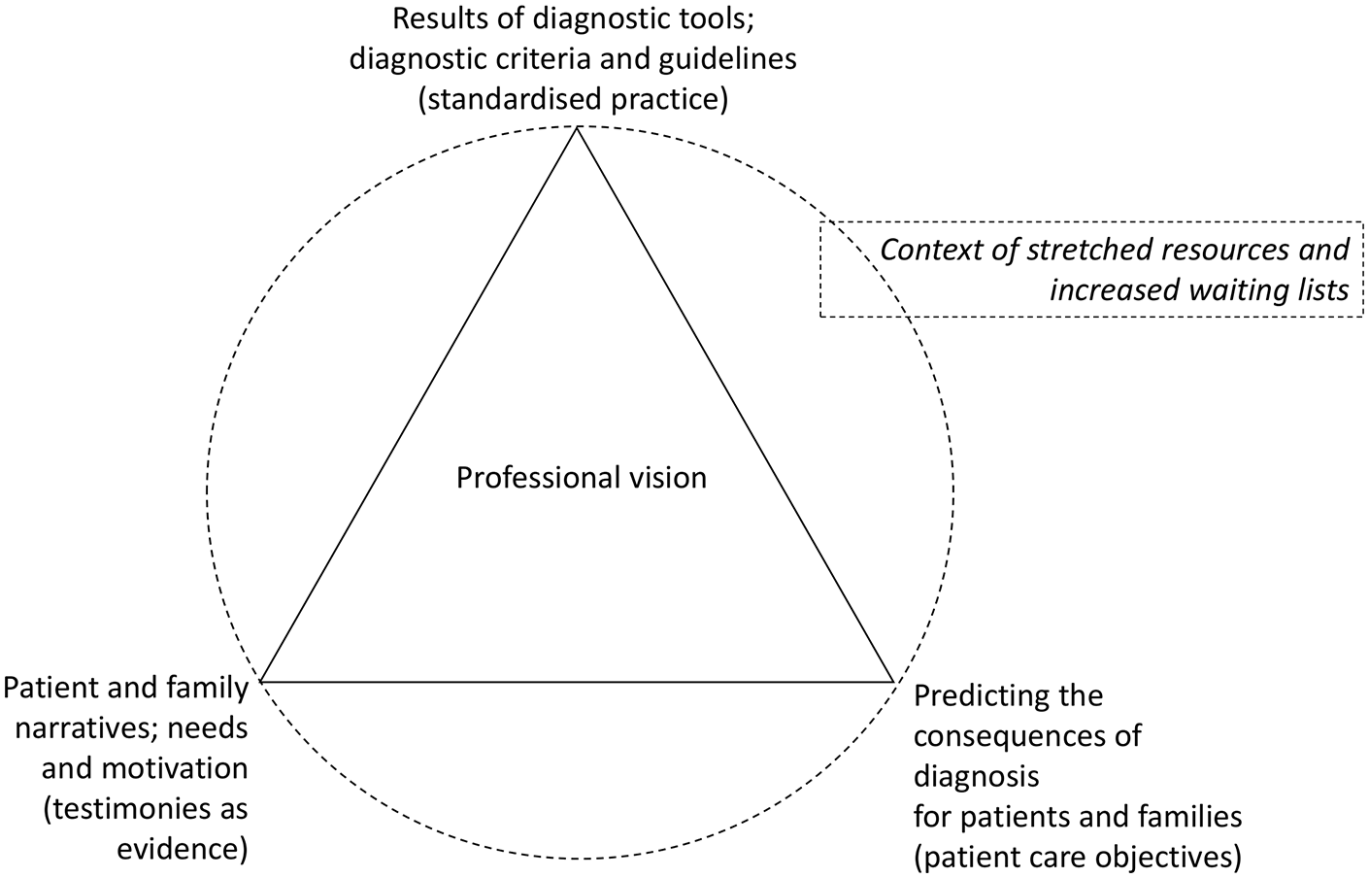
Aligning or conflicting interests in diagnostic practice.

To make a decision, clinicians must decide together whether tensions align or conflict, and find a way to resolve them. In this study, clinicians are pulled between the institutional requirements outlined in diagnostic manuals and facilitated through diagnostic tools (standardised practice), the interpretative narrative that patients and families deliver in the assessment process (testimonies as evidence) and the perceived consequences of diagnosis (or not) on patients and families in their charge (patient-care objectives) (see Figure 2). These tensions are framed within a context of stretched resources and increased waiting lists. Decisions about whether these are aligned or conflicting are resolved by clinicians through their professional vision, which exists through the tools they use, and draws on clinical judgement, experience and knowledge. In this case, professional vision is a set of professionally defined, practice-led, interpretative practices that then become, in themselves, standardised practice.

## Strengths and limitations

The TAR method facilitated discussion about specific cases, enabling reflection by participants on the process and acting as an aide-mémoire. This allowed a rich and free-flowing discussion in interview. However, as cases are pre-selected by the researcher, the issues discussed will be shaped by that selection. A limitation of the study was that participants were primarily self-selecting, and were involved in interview only if part of a team taking part in a wider observation study. There was no opportunity, therefore, to ensure a full breadth of professional roles, ages or ethnic and geographical diversity.

## Concluding comments

We set out to explore how autism diagnosis could be considered a social process shaped by wider social forces and interaction (Brown, 1995) as described by clinicians engaged in assessment. Our findings suggest that autism diagnosis is not a straightforward, linear, clinical process, but rather one which is, in itself, a socially situated activity. The clinical implications of this are threefold. First, a greater understanding of the complexity of assessment may help ‘translation’ of diagnostic communication between clinicians and patients. This may ease some of the tensions felt by patients and families coming for diagnosis which are created through the system itself, leading to what might be termed an ‘enhanced alliance’ between parties (Jacobs, Hens, et al., 2019, p. 502). This includes the understanding that diagnosis is not only about ‘scoring’ on specialist tests. Second, we support other research which identifies that the process of autism assessment in England is the one that does not currently fully meet the needs of patients and families, and this pressure is also felt by clinicians. To fulfil the assessment requirements outlined in autism strategies and clinical guidelines, further resources need to be allocated. Finally, we conclude that while the rationale for creating a specialist autism pathway has benefits in terms of clarity of process and development of clinical specialisms, some clinicians have suggested that this encourages a ‘narrow lens’ rather than an examination of broader needs. A separation from broader health assessments, it is argued, can have a negative effect in inhibiting a holistic approach to assessing an individual for a range of complex needs. We suggest that an examination of the benefits and drawbacks of assessment services specialising in autism only, the resources they require to operate effectively and how they operate in the context of wider health services would be appropriate and timely.

## Supplemental Material

sj-pdf-1-aut-10.1177_13623613211030392 – Supplemental material for Autism diagnosis as a social process Supplemental material, sj-pdf-1-aut-10.1177_13623613211030392 for Autism diagnosis as a social process by Jennie Hayes, Tamsin Ford, Rose McCabe and Ginny Russell in Autism
