# Supplementary material for: Autism diagnosis as a social process
Source: Autism. 2021 Jul 9;26(2):488–98. doi: 10.1177/13623613211030392 (PMC8814948; doi:10.1177/13623613211030392)
Supplement: sj-pdf-1-aut-10.1177_13623613211030392 – Supplemental material for Autism diagnosis as a social process [file sj-pdf-1-aut-10.1177_13623613211030392.pdf]

Supplemental file: Interview strategy and Topic Guide

Research Question: What are the social factors that drive diagnostic practice?

Overview: follow up interviews with clinicians involved in MDT meetings will, where possible, explore the trajectory of particular cases raised in MDT meetings and elicit clinicians' perspectives around specific influences on decision-making, drawn from the MDT data, enabling detailed exploration of the mechanisms behind decision-making with those taking part.

Sampling: interviews will be conducted with healthcare professionals (HCPs) involved in the MDTs observed, between 15 and 20 interviews.

Method: A semi-structured approach will be used and the order of questions may vary depending on the progress of the interview. The first two parts of the interview comprise a series of questions. Part three of the interview will draw from a case which has been discussed at a team meeting (tape assisted recall). Where possible, a transcript of the meeting will be available, along with the audio recording. This case will be selected on the basis of an emerging social factor drawn from the data (e.g. parental/informant/patient desire driving process; need/functionalist approach or clinical approach; masking of symptoms/insight or performing autism). Where possible, extracts from the audio recording and/or transcript will be used as prompts during the interview. Where this is not possible, cases will be discussed as examples without prompts.

Analysis: Interviews will be audiotaped, transcribed and analysed with thematic analysis. This will enable broad themes to be summarized from the data suited to informing policy development (Braun & Clarke, 2006). The questions below will function as prompts for interview. Follow up questions and discussion will arise from the individual interview and may vary or develop in different directions.

Interview schedule

Background and administrative items: interviewer explains purpose and context of interview, length and structure, right to withdraw, confidentiality and audio recording. Participant completes consent form and demographic info.

Part one: the diagnostic process

1. To start with, can you talk about the different things you take into account during diagnosis?
2. How does the MDT meeting contribute to dx process?
3. How do different roles in meetings contribute to negotiating the diagnostic decision?
4. How do factors outside your control impact on dx: eg SES, clinician availability, resources, govt spending etc, waiting lists?
5. How do you deal with that intersection between the social/environmental/biological/behavioural aspects of autism?

Part two: using diagnostic tools and guidelines

1. How do you use clinical guidelines? (and which ones) ICD/DSM etc and or NICE etc.
2. How do you use diagnostic instruments (and which ones?)
3. How do they contribute to dx process?

Part three: case discussion drawing from MDT data – tape/transcription assisted recall

1. Can you tell me a bit about NAME?
2. Can you tell me where they were referred from?
3. Do you know the background to them seeking a diagnosis?
4. Specific questions to prompt based on the data extract
5. Is there anything you'd like to add?

Debrief: Interviewer will bring the interview to a close, thank participants for their involvement, describe next steps for research project and how they can keep in touch.

Braun, V., & Clarke, V. (2006). Using thematic analysis in psychology. *Qualitative Research in Psychology, 3*(2), 77–101. https://doi.org/10.1191/1478088706qp063oa
